# Cerebral Microvascular Senescence and Inflammation in Diabetes

**DOI:** 10.3389/fphys.2022.864758

**Published:** 2022-04-29

**Authors:** Ashley Phoenix, Raghavendar Chandran, Adviye Ergul

**Affiliations:** ^1^ Department of Pathology and Laboratory Medicine, Medical University of South Carolina, Charleston, SC, United States; ^2^ Ralph H. Johnson Veterans Affairs Medical Center, Charleston, SC, United States

**Keywords:** cerebral vasculature, vascular, senescence, cognitive impairment, diabetes

## Abstract

Stress-induced premature senescence can contribute to the accelerated metabolic aging process in diabetes. Progressive accumulation of senescent cells in the brain, especially those displaying the harmful inflammatory senescence-associated secretory phenotype (SASP), may lead to cognitive impairment linked with metabolic disturbances. In this context, the senescence within the neurovascular unit (NVU) should be studied as much as in the neurons as emerging evidence shows that neurogliovascular communication is critical for brain health. It is also known that cerebrovascular dysfunction and decreased cerebral blood flow (CBF) precede the occurrence of neuronal pathologies and overt cognitive impairment. Various studies have shown that endothelial cells, the major component of the NVU, acquire a senescent phenotype via various molecular mediators and pathways upon exposure to high glucose and other conditions mimicking metabolic disturbances. In addition, senescence in the other cells that are part of the NVU, like pericytes and vascular smooth cells, was also triggered upon exposure to diabetic conditions. The senescence within the NVU may compromise functional and trophic coupling among glial, vascular, and neuronal cells and the resulting SASP may contribute to the chronic neurovascular inflammation observed in Alzheimer’s Disease and Related Dementias (ADRD). The link between diabetes-mediated cerebral microvascular dysfunction, NVU senescence, inflammation, and cognitive impairment must be widely studied to design therapeutic strategies.

## 1 Introduction

Senescence is a biological process defined by an apoptosis-resistant and irreversible arrested cell cycle with a distinct pro-inflammatory phenotype affecting neighboring cells (Hayflick and Moorhead, 1961; Rodier and Campisi, 2011; Acosta et al., 2013; Childs et al., 2015; He and Sharpless, 2017). Accumulation of senescent cells in various tissues is believed to contribute to progressive functional impairments that come with chronological aging as well as with age-related disorders such as Alzheimer’s Disease and Related Dementias (ADRD) including Vascular Contributions to Cognitive Impairment and Dementia (VCID) (Sikora et al., 2021a). Senescent inflammatory phenotype can also be triggered by metabolic disturbances as seen in diabetes and obesity, major comorbidities for individuals suffering from ADRD, and led to the concept of premature metabolic aging (Palmer et al., 2015; Burton and Faragher, 2018). Emerging evidence suggests that the removal of senescent cells by pharmacological or genetic approaches improves cognitive deficits in animal models of ADRD (Bussian et al., 2018; Kim and Kim, 2019; Zhang et al., 2019). However, our understanding of the role of senescence in healthy brain aging, and what tips the scale to pathological senescence is in its infancy. The landscape of senescence among brain cells is unclear. Delineation of the molecular and cellular mechanisms and functional consequences of senescence may lead to the design and implementation of effective therapies.

It is established that cerebrovascular dysfunction and decreased cerebral blood flow (CBF) are early findings preceding the development of neuronal pathologies such as amyloid and tau deposition as well as cognitive deficits (Gorelick et al., 2017). The brain is a metabolically demanding organ that requires constant blood flow to perform all the complex functions ranging from cognition to regulation of cardiovascular homeostasis. As such, it has a dense microvascular network, and endothelial cells which form the neurovascular unit (NVU) along with the surrounding glial cells and pericytes play very critical roles in the delivery of nutrients, removal of waste, and formation of blood brain barrier (BBB) (Gorelick et al., 2017; Iadecola, 2017; Sweeney et al., 2019a; Sweeney et al., 2019b). Endothelial senescence can trigger a vicious inflammatory cycle and disrupt brain homeostasis. Thus, in this review, we will first summarize the current understanding of the senescence process, and its physiological and pathophysiological consequences in general. Next, we will review the role of NVU senescence in cognitive impairment in the context of diabetes.

## 2 Evolving Concept of Cellular Senescence Phenotypes

**FIGURE 1:**
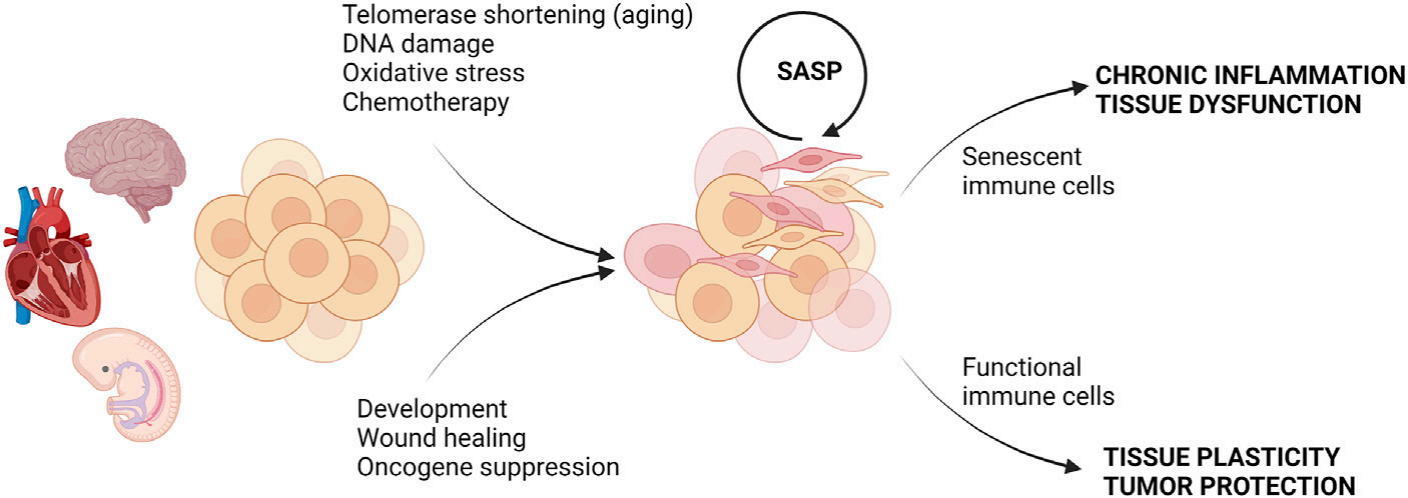
Consequences of cell senescence in health and disease. Different types of stress or physiological conditions can trigger senescence. In embryonic development and tissue repair, senescence is beneficial and clearance of senescent cells by functional immune cells restores tissue function. In contrast, the accumulation of senescent cells under stress conditions can be further exacerbated by SASP, and failure of the removal of senescent cells by senescent immune cells can lead to chronic inflammation and tissue dysfunction. Created with BioRender.com.

When the idea of cell senescence was first introduced in 1961 by Hayflick and Moorhead, it was thought to be a normal process of aging (Hayflick and Moorhead, 1961). However, in the years since its discovery, it has been proven that cellular senescence can be both a typical physiological process and in other instances deleterious to the homeostasis of the surrounding cellular environment (Childs et al., 2015; He and Sharpless, 2017; Rodier and Campisi, 2011; Acosta et al., 2013) (Figure 1). While senescence can be beneficial in embryonic development, tissue repair, and tumor suppression, accumulation of senescent cells and their products can promote chronic inflammation, tissue damage, and impairment of tissue regeneration (He and Sharpless, 2017; Rodier and Campisi, 2011; Acosta et al., 2013). It is now recognized that the senescent phenotype is heterogeneous and can be categorized as replicative senescence, stress-induced premature senescence (SIPS), and oncogene-induced senescence (Figure 1). In the following section, we briefly review replicative senescence and SIPS as they relate to chronological and metabolic aging, respectively. We refer the readers to excellent recent review articles that describe the molecular processes and the detailed characterization of senescent phenotypes (Gorgoulis et al., 2019; Sikora et al., 2021b).

### 2.1 Replicative Senescence

**FIGURE 2:**
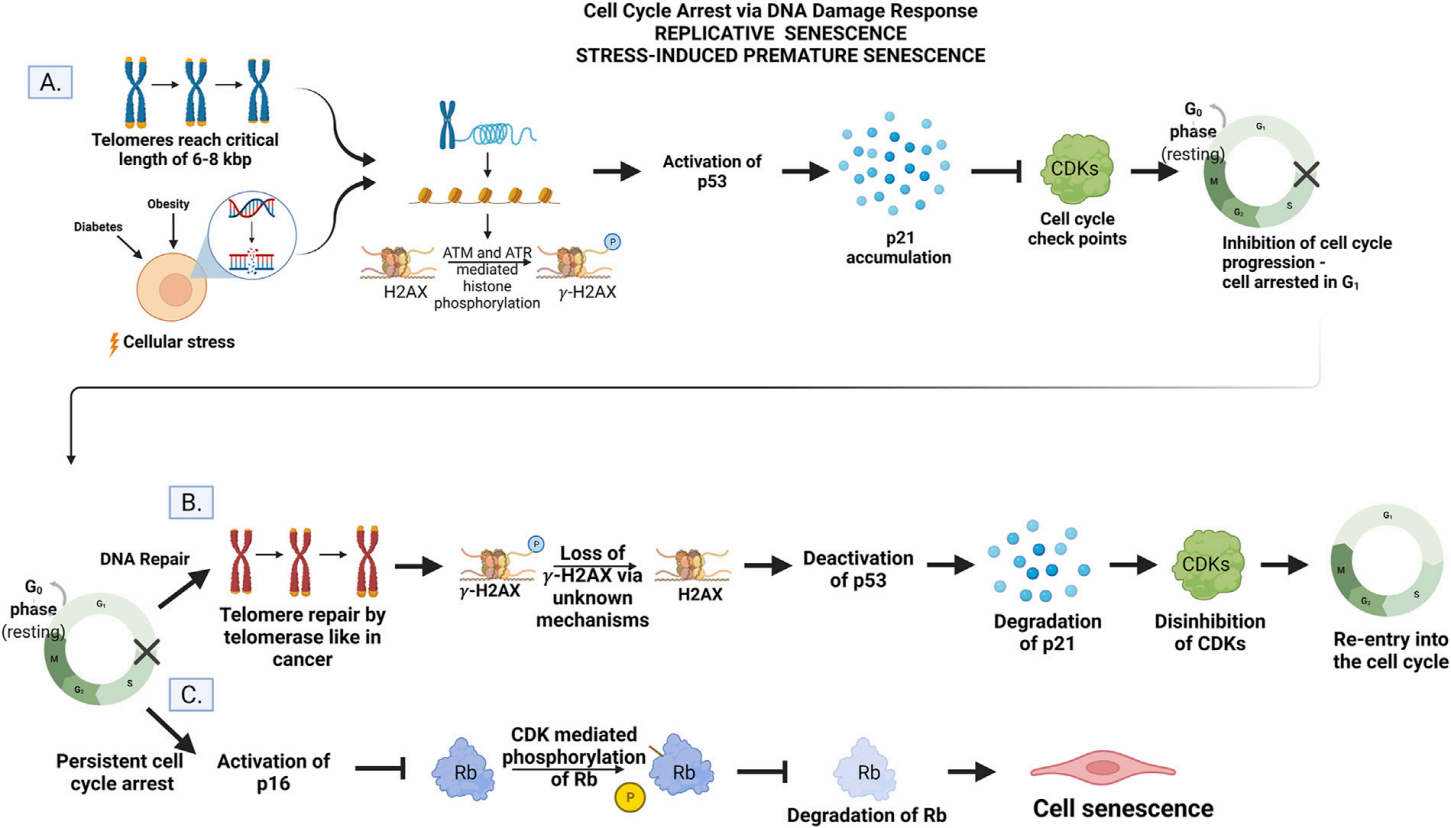
**(A)** Cell cycle arrest. Telomere shortening to the critical length of 6–8 kbp or cellular stress activates ATM and ATR to mediate histone phosphorylation of H2AX to gamma-H2AX. The accumulation of gamma-H2AX activates p53 and upregulates the gene p21. Inhibition of CDKs by p21 prevents progression of the cell cycle conditionally. **(B)** DNA repair and re-entry into the cell cycle. The addition of DNA to telomere ends by telomerase instigates the loss of histone phosphorylation via unknown mechanisms. The p53/p21 pathways are then deactivated, p21 is degraded, and CDKs are disinhibited allowing the cell to re-enter and progress through the cell cycle. **(C)** Persistent cell cycle arrest leads to senescence. The DNA damage is unable to be repaired by the cell leading to the activation of the p16 gene. Phosphorylation and subsequent degradation of Rb by CDKs are inhibited. Rb activity instigates permanent exit from the cell cycle but not cell death (senescence) via unknown mechanisms. Created by with BioRender.com.

Senescence is when a cell is irreversibly arrested in the cell cycle, and therefore, it is no longer able to replicate but remains metabolically active (Palmer et al., 2015; Sikora et al., 2021b). The primary physiological trigger of senescence is irreparable DNA damage that is most often linked to the DNA Damage Response (DDR) (Figure 2). This can also be observed in the normal processes of cellular aging. After a finite number of replications known as the “Hayflick limit”, the telomeres, a portion of non-coding DNA at the end of each chromosome meant to protect the integrity of DNA strands, shortens to a critical length (Hayflick and Moorhead, 1961; Liu et al., 2019). After this point, the ends of the DNA are identified as strand breaks by internal cellular mechanisms, and DDR is initiated triggering cell senescence via ataxia telangiectasia mutated (ATM) and ataxia telangiectasia and RAD3-related (ATR). These kinases are responsible for the accumulation of the cell cycle inhibitor protein-53 (p53) (Herbig et al., 2004; Mu et al., 2011; Burton and Faragher, 2015; Sikora et al., 2021b). Herbig and colleagues showed that at the critical length of 6–8 kbp, the telomeres show a collection of H2AX histones that are phosphorylated to the variant γ-H2AX in human fibroblasts. This phosphorylation, regarded as a marker of DNA damage, is mediated by ATM and ATR and represents the fact that these telomeres are now seen as double-strand breaks (DBS) and considered to be damaged DNA by the cell (Herbig et al., 2004). This prevents the cells from continuing to replicate until the telomeres are entirely gone, and the DNA would no longer be copied in its entirety. This phenomenon impedes mutations that would result from incomplete DNA replication and is termed *replicative senescence* (Liu et al., 2019). In this context, replicative senescence can be viewed as a protective phenomenon.

This type of senescence is also thought to be a normal process of aging due to the strong correlation between age and increased senescence markers (Gorgoulis et al., 2019; Sikora et al., 2021b). It is initiated by the p53/p21 pathway where p53 upregulates the gene p21 which, in turn, inhibits the function of cyclin-dependent kinases (CDKs) required for the cell to progress through the cell cycle (Figure 2) (Sikora et al., 2021b). As the telomeres shorten, the damage becomes persistent, and cells undergo permanent arrest (Rodier and Campisi, 2011; He and Sharpless, 2017). In this context, replicative senescence is regarded as a chronological aging phenomenon.

### 2.2 Stress-Induced Premature Senescence

Senescence can also be triggered by repeated insults to the cell via disease pathologies as seen in diabetes and obesity (Palmer et al., 2015; Burton and Faragher, 2018). This is known as SIPS and is independent of telomere length, but reliant on constitutive DDR signaling to activate ATM and ATR (Sikora et al., 2021b). These same proteins p53, p21, and p16 are also responsible for arresting the cell cycle in response to pathological DNA damage after promotion by ATM and ATR. However, SIPS can also be instigated via p16 independent of the p53/p21 pathway (Burton and Krizhanovsky, 2014; Burton and Faragher, 2018; Kim et al., 2022). This can be initiated in response to increased levels of reactive oxygen species (ROS), cell-cell fusion, and various other adverse stimuli like UV irradiation (Mu et al., 2011; Palmer et al., 2015; Sikora et al., 2021b; Wiley and Campisi, 2021). In this context, SIPS is regarded as a premature aging event.

### 2.3 Cellular Consequences

#### 2.3.1 Beneficial Effects

The benefit of cellular senescence is that cells with damaged DNA cease replication, thereby reducing the opportunity for mutations that could be potentially harmful to the organism, such as cancer (Burton and Krizhanovsky, 2014; Sikora et al., 2021b; Wiley and Campisi, 2021). Without the induction of senescence, for example, after UV irradiation resulting in single-strand DNA breaks in murine embryo, the organism ultimately succumbed to the DNA mutations which proved to be fatal (Mu et al., 2011). In this way, by not simply undergoing apoptosis or some other form of immediate cell death or removal from the environment, the organism can maintain not only the integrity of the tissue until new cells can be produced but also the viability of the organism (Burton and Krizhanovsky, 2014; He and Sharpless, 2017; Gorgoulis et al., 2019). Cell senescence is also a favorable process in wound healing and embryonic development (Burton and Krizhanovsky, 2014; He and Sharpless, 2017; Gorgoulis et al., 2019) (Figure 1). This again highlights the protective potential of cell senescence.

#### 2.3.2 Detrimental Effects

When senescent cells accumulate, they can become damaging. Cells can undergo phenotypic changes such as flattening, stochastic gene expression thought to derive from chromatin remodeling, altered paracrine activity, and increased production of reactive oxygen species (Burton and Krizhanovsky, 2014; Burton and Faragher, 2015; He and Sharpless, 2017; Gorgoulis et al., 2019). Senescence can also lead to cellular metabolic changes with deleterious consequences. For example, human hematopoietic stem and progenitor cells as well as human fibroblasts have been shown to display increased glucose uptake and glycolysis, and lipid accumulation (Gorgoulis et al., 2019; Poisa-Beiro et al., 2022). This alteration in lipid handling is detrimental in that it accumulates within lysosomes as indigestible lipofuscin, impairing lysosomal function and autophagic capabilities. In this regard, the relationship between autophagy and senescence is bidirectional (Rajendran et al., 2019; Wiley and Campisi, 2021). Impaired autophagy can promote and exacerbate senescence via the promotion of *senescence-associated secretory phenotype (SASP),* which is further discussed below. In addition to dysregulation of lysosomal function and lipid metabolism, altered transition metal homeostasis is another example of metabolic changes in senescent cells (Masaldan et al., 2018a; Masaldan et al., 2018b). For detailed information, we refer the readers to a recent review on the interplay between metabolism and senescence (Wiley and Campisi, 2021). These changes in cell morphology and metabolism as well as gene expression prevent the cell from carrying out its intended processes which could potentially lead to tissue dysfunction and compromised tissue regeneration as senescent cells amass over time (Burton and Krizhanovsky, 2014; Burton and Faragher, 2015; He and Sharpless, 2017; Gorgoulis et al., 2019). For instance, Minamino et al. showed that senescent human aortic endothelial cells displayed decreased endothelial nitric oxide synthase (eNOS) activity. With an accumulation of cell senescence in this area, the vasorelaxation capability of the arteries can be impaired leading to atherosclerosis (Minamino and Komuro, 2002).

The most impactful behavior of senescent cells is their secretory phenotype (Acosta et al., 2013; Tasdemir and Lowe, 2013; Burton and Krizhanovsky, 2014; Burton and Faragher, 2015; Gorgoulis et al., 2019; Sikora et al., 2021b). Senescent cells can secrete a host of inflammatory cytokines, chemokines, growth factors, and enzymes known as SASP or the senescence secretome. SASP is a complex feature of senescence. While it is common to all senescent phenotypes, the composition of SASP is very diverse depending on the cause and cell type (Freund et al., 2010; Rodier and Campisi, 2011; Wiley and Campisi, 2021). As a result, the regulation and consequences of SASP are also complex (Acosta et al., 2013; Tasdemir and Lowe, 2013; Burton and Krizhanovsky, 2014; Burton and Faragher, 2015; Gorgoulis et al., 2019; Sikora et al., 2021b).

In a healthy model, the upregulation of these molecules as well as cell surface proteins like NKG2, an excitatory ligand for natural killer cells, allows for the localization of the senescent cells by the immune system and their subsequent clearance. The upregulation of NKG2, in specific, allows for the recognition and elimination of the senescent cells by natural killer cells via granule exocytosis (Burton and Krizhanovsky, 2014; Burton and Faragher, 2015; Sagiv et al., 2016; Gorgoulis et al., 2019; Sikora et al., 2021b). Senescent cells can also be eliminated by monocytes/macrophages, T, and B cells (Sagiv et al., 2016; Sikora et al., 2021b).

The harmful impact of senescent cells arises when there is a failure for their removal by immune surveillance. This can happen as a result of an aged immune system that is no longer able to accurately identify and remove the cells, or by the accumulation of senescent cells at a rate too high for the immune system to clear. Sirtuin1/SIRT1 [(*silent mating type information regulation 2 homolog*) *1 (S. cerevisiae)*], has been observed to be decreased in aged immune CD4^+^CD28^−^ T cells of humans as well as in aged mice models and has been implicated as a potential target for slowing this immune aging (Xu et al., 2020). As a result, SASP leads to persistent sterile inflammation that is damaging to the neighboring cells and propagates cell senescence in a paracrine manner (Acosta et al., 2013; Tasdemir and Lowe, 2013; Palmer et al., 2015). Diverse SASP factors promoting inflammation include transforming growth factor (TGF)-β, IL-1, IL-6, IL-17, monocyte chemoattractant protein (MCP)-1, tumor necrosis factor (TNF)-α, insulin-like growth factor (IGF)-1, and matrix metalloproteases (MMP) among others (Admasu et al., 2021). Central to this review, studies have shown upregulation of these factors and adhesion molecules in endothelial and vascular smooth muscle cells of the cerebral microvasculature in rodent models perpetuate vascular inflammation (Ungvari et al., 2013; Fulop et al., 2018; Ungvari et al., 2018), which is further discussed in the next section.

## 3 Cellular Senescence Within the Brain and Cognitive Impairment

In the context of brain health, our limited understanding of the role of senescence in healthy brain aging is a barrier to delineating the complex role of senescence in neurodegenerative diseases. Aging-related cognitive decline is not entirely an outcome of neuronal death. Neuronal senescence can contribute to its pathophysiology (Hof and Morrison, 2004). As discussed above, when brain cells enter into a “senescent state” characterized by permanent cell cycle arrest, apoptosis-resistance, altered gene expression, and inflammatory secretory phenotype (Tasdemir and Lowe, 2013; Burton and Krizhanovsky, 2014; Gorgoulis et al., 2019; Kim and Kim, 2019), they can propagate this signal to neighboring cells. There is evidence that neurons, terminally differentiated cells, can acquire a senescence-like state (Geng et al., 2010; Jurk et al., 2012; Piechota et al., 2016). However, whether neuronal senescence occurs and leads to neurodegenerative diseases is still under investigation (Sikora et al., 2021a). Elucidating the functional and physiological consequences of senescence by cell types in the brain during aging could uncover new targets for neurodegeneration and subsequent cognitive decline. Given that nitrative/oxidative stress is a major inducer of senescence, and diabetes being an inflammatory disease associated with exacerbated oxidative/nitrative stress and premature vascular aging, we will focus on the role of neurovascular unit senescence in cognitive impairment linked to diabetes. We refer the readers to an excellent recent review on brain aging and senescence (Sikora et al., 2021a).

### 3.1 Diabetes and Cognitive Impairment

**FIGURE 3:**
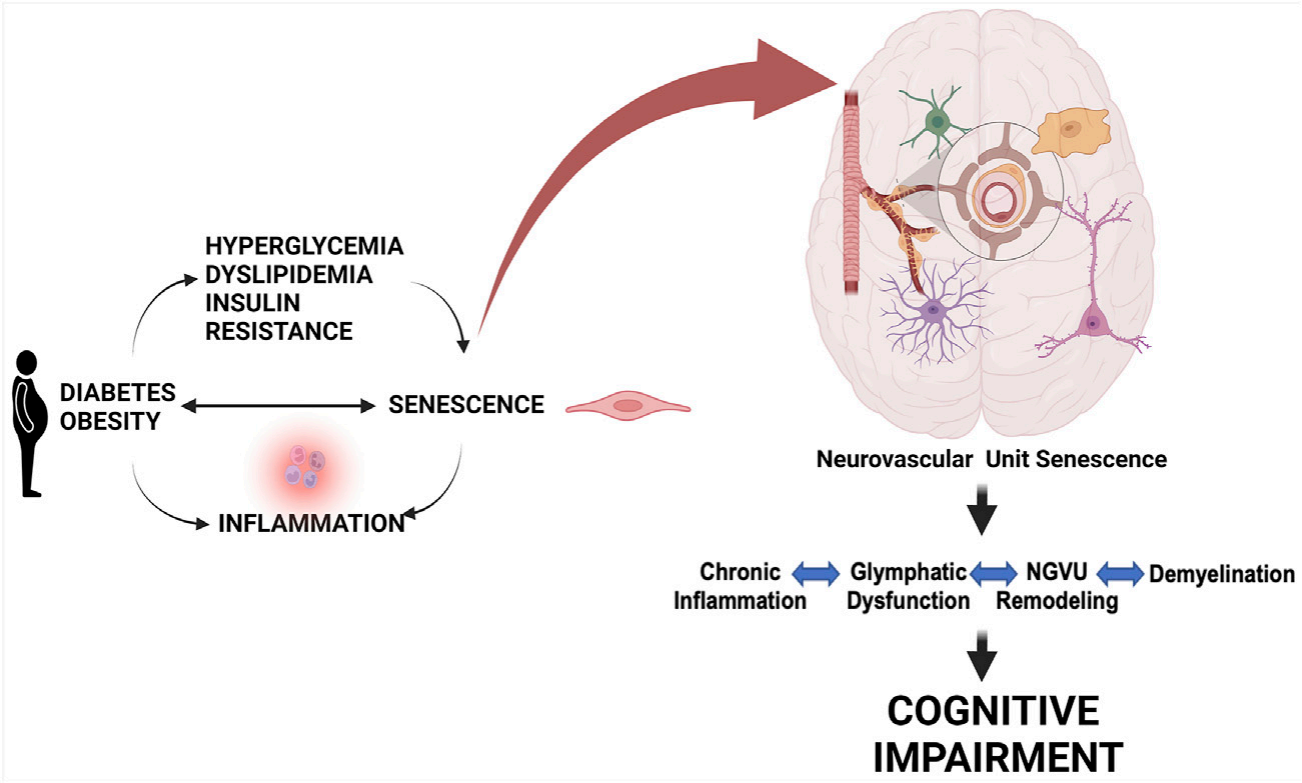
Role of cell senescence in metabolic disease-associated cognitive impairment. Metabolic diseases including obesity and diabetes and conditions linked to them like hyperglycemia, dyslipidemia, and insulin resistance can lead to a senoinflammatory loop. Senescence of pancreatic cells can also lead to diabetes. This persisting senescence at the cellular level targeting the components of the NGVU gradually leads to chronic inflammation, glymphatic dysfunction, NGVU remodeling, and demyelination resulting in progressive cognitive impairment. Created with BioRender.com.

Brain health is dependent upon tightly regulated CBF and BBB integrity by a well-coordinated cell-cell interaction in this network (Demuth et al., 2017; Schaeffer and Iadecola, 2021). Epidemiological studies have long established the coexistence of cognitive deficits, ranging from cognitive dysfunction to dementia, and diabetes in older adults (Cheng et al., 2012). Only in recent years, it has become clearer that cognitive deficits might be a direct complication of diabetes, shifting from correlation to causation (Biessels et al., 2020; Srikanth et al., 2020; van Sloten et al., 2020). Cerebrovascular dysfunction is a common pathology between diabetes and dementia (Li W. et al., 2018). Endothelial dysfunction and decreased CBF are early changes that precede the development of neuropathologies (tau and amyloid deposition) and cognitive deficits (Gorelick et al., 2017; van Sloten et al., 2020). The endothelium is an early target in metabolic diseases including diabetes. Cerebrovascular dysfunction resulting from an imbalance of endothelium-derived vasoconstrictor and vasodilator substances can contribute to the early decrease in CBF, which could trigger oxidative/nitrative stress initiating a senoinflammatory loop (Figure 3). The human brain has about 400 miles long vascular network, most of which is formed by capillaries lined with endothelial cells (Begley and Brightman, 2003; Coucha et al., 2013; Kiss et al., 2020). Since high glucose conditions are known to initiate premature cellular senescence, and endothelial cells are the first line of barrier exposed to hyperglycemia as well as being the interface between the brain and blood (Chen et al., 2002; Biessels and Despa, 2018; Prattichizzo et al., 2018; Balasubramanian et al., 2021), in the following section we will first review evidence on senescence in endothelial cells followed by other cells of the NVU in diabetes.

### 3.2 Endothelial Senescence in Diabetes and Metabolic Diseases

The bulk of our knowledge comes from *in vitro* studies in which high glucose was used to mimic diabetes using non-CNS vascular endothelial cells, especially in the widely known human umbilical vein endothelial cells (HUVECs) which are not ideal endothelial cells explaining adult disease conditions as opposed to brain microvascular endothelial cells that are at the center of the NVU. Evidence has conclusively shown that the high glucose insult completely changes the phenotype of the endothelial cells (Senthil et al., 2017; Prattichizzo et al., 2018; Haspula et al., 2019), which is also pro-apoptotic (Senthil et al., 2017). Widely known modulators of cellular senescence like SIRT1 and p53 have been found to play an important role in diabetes-linked premature vascular senescence. HUVECs exposed to high glucose conditions express increased levels of Src homology/collagen (Shc) adaptor protein p66Shc which plays a crucial role in increased ROS production. SIRT1 overexpression was shown to inhibit this harmful increase in p66Shc levels in HUVECs as well as protection against high glucose–induced endothelial dysfunction indicated by the increase in manganese superoxide dismutase (MnSOD) and decrease in plasminogen activator inhibitor-1 (PAI-1) expression. Also, SIRT1 levels were found to be decreased in the aorta of diabetic mice compared to normal controls. SIRT1 overexpression in the aorta of diabetic endothelium-specific SIRT1 transgenic (SIRT1-Tg) mice led to a decrease in p66Shc, nitrotyrosine, and 8-OHdG levels compared to diabetic wild type mice (Zhou et al., 2011). In a follow-up study, the same authors have observed decreased levels of senescent markers like p53, p21, and PAI-1 as well as p66Shc in the aorta of diabetic mice with endothelium-specific SIRT1-Tg, compared with diabetic wild type mice (Chen et al., 2012). Senthil et al. have shown that the activation of nuclear factor erythroid 2–related factor 2 (Nrf2)-mediated antioxidant genes prevent hyperglycemia-induced senescence and apoptosis in HUVECs (Senthil et al., 2017).

Advanced glycated end products (AGEs) are an outcome of the chronic hyperglycemia state by a non-enzymatic glycation process involving glucose and mostly proteins but also lipids in some cases. They are known to play a role in vascular complications associated with diabetes (Rhee and Kim, 2018). HUVECs treated with glycated collagen initiates a premature senescent phenotype found to be a consequence of a decrease in NO availability with a concomitant increase in peroxynitrite and superoxide levels (Chen et al., 2006). Premature senescent cells were also found to be increased in the aorta of young Zucker diabetic rats as compared to controls (Tsuboi et al., 2018). Treatment with ebselen (peroxynitrite scavenger), NOHA (intermediate eNOS Substrate), and SOD mimetic MnTBAP reversed the premature senescence in GC-treated HUVECs indicating a link between the premature senescence and the reduced NO and increased peroxynitrite/superoxide levels (Chen et al., 2006). The same group reported increased senescent cells and impaired endothelial dysfunction in the aorta of diabetic rats which were reversed with ebselen treatment (Brodsky et al., 2004). A recent genetic profiling study has shown cellular senescence as one of the major signaling pathways that is significantly affected in the neurons of the brains of type 2 diabetic patients in which endothelial cells showed the robust formation of AGEs (Bury et al., 2021).

The increase in the p53 transcription factor is known to play a major role in the upregulation of cellular senescence (Gorgoulis et al., 2019; Sikora et al., 2021b). Endothelial p53 is increased in diabetic mice which was associated with impaired vasodilatation and p53 knockout reversed this harmful outcome (Yokoyama et al., 2019). Inhibition of dipeptidyl-peptidase 4 (DPP-4) in diabetic fatty rats reduced senescence along with an increase in the levels of glucagon-like peptide 1 (GLP-1), an antidiabetic hormone, providing additional evidence for increased senescence in diabetes. In parallel, GLP-1 treatment was shown to be inhibiting stress-induced senescence in HUVECs, thus validating their anti-senescence activity (Oeseburg et al., 2010).

Endothelial progenitor cells (EPCs) are considered to be a biomarker/cellular surrogate for endothelial health/function and play a possible role in the management of cerebrovascular diseases (Lapergue et al., 2007). EPCs exposed to high glucose conditions *in vitro* led to an increased senescent phenotype which was reversed by treatment with a p38 MAPK inhibitor (Kuki et al., 2006). Atherogenic dyslipidemia is an important characteristic of diabetes mellitus and an outcome of factors like the increase in small dense low-density lipoprotein particles (Poznyak et al., 2020). Rosso et al. have shown in an interesting study that circulating EPCs from normal donors exhibited senescent characteristics when cultured in the presence of oxidized small and dense low-density lipoprotein (ox-dmLDL) from diabetic donors and the Akt/p53/p21 signaling pathway was found to play the main role in this transition (Rosso et al., 2006). We refer the readers to an excellent recent review article that discusses the metabolic roots and bidirectional interaction of senescence with metabolic diseases (Wiley and Campisi, 2021).

Evidence for senescence in brain endothelial cells is relatively limited. While it is not under diabetic conditions, a few studies suggested microvascular/endothelial senescence. A recent study showed greater expression of genes associated with senescence in the microvessels isolated from postmortem brains of AD patients (Bryant et al., 2020). Another study reported the role of endothelial SASP in contributing to cerebrovascular disease pathology through an *in vitro* study investigating the molecular basis for the onset of cerebral cavernous malformations (CCMs), a widely studied rare genetic disease but with no etiology to metabolic factors. Vannier *et al.* reported the loss of CCM1 and CCM2 in endothelial cells (HUVECs) triggers them to transition to a senescence-associated secretory phenotype via activation of Rho-associated coiled-coil containing protein kinase 2 (ROCK2). The authors had also proposed that the SASP changes the tissue microenvironment by increasing extracellular matrix degradation (Vannier et al., 2021). Kiss and colleagues identified senescent cerebromicrovascular endothelial cells in the aged mouse brain using complex analyses of single-cell RNA sequencing data (Kiss et al., 2020). These reports highlight the need for additional studies to investigate the role and mechanisms by which cerebral endothelial senescence contributes to neurovascular inflammation in aging and disease states.

Evidence for senescence in brain endothelial cells in diabetes or diabetes-like conditions is even more limited. Retinal microvasculature and endothelial cells are considered a window to brain microvasculature. Oxidative and nitrative stress has been shown to accelerate the aging of the retinal vasculature in diabetes (Lamoke et al., 2015). Another study reported that NADPH oxidase 2 (NOX2)-induced increases in arginase 1 (A1) activity promotes premature senescence of retinal endothelial cells in streptozotocin-induced diabetes (Rojas et al., 2017). A follow-up study showed that genetic or pharmacological blockade of A1 prevents retinal endothelial senescence in diabetic mice (Shosha et al., 2018). Moreover, SASP cytokines are found to be elevated in vitreous samples from patients with proliferative diabetic retinopathy (Oubaha et al., 2016). Given the alarming increase in the incidence of diabetes and the critical role of brain endothelial cells in brain health, there is a need to further investigate brain endothelial cell senescence across the cerebrovascular bed in diabetes.

### 3.3 Mural Cell Senescence in Diabetes and Metabolic Diseases

The majority of research on metabolic disease-induced vascular diseases is focused on the endothelial component. As discussed above, even brain endothelial cell senescence in diabetes is poorly understood but it is imperative to reiterate that the overall NVU health plays a major role in the pathophysiology of cognitive impairment in the context of metabolic disease. Pericytes and vascular smooth cells (VSMCs) are the other major cells of the cerebrovascular wall that will be discussed next.

Pericytes are embedded in the basement membrane and coordinate signals between NVU cells (Sweeney et al., 2016; Brown et al., 2019) to regulate CBF, BBB development and integrity, angiogenesis, and waste clearance from the brain. While pericytes were discovered in the 19th century, they were considered the support cells, and the critical roles they play in the development and progression of cognitive impairment as well as other neurodegenerative diseases are being recognized only in recent years as elegantly reviewed in these recent papers (Lendahl et al., 2019; Ding et al., 2020; Uemura et al., 2020; Bennett and Kim, 2021). Technological advances significantly contributed to these developments (Berthiaume and Shih, 2019). An interesting paper to highlight is the single-cell analysis of BBB response to pericyte loss which provided novel and intriguing information on the complexity of endothelial cell responses to pericyte deficiency (Mae et al., 2021).

While a majority of the studies report pericyte dysfunction in cognitive impairment and neurodegenerative diseases, pericyte senescence, let alone in diabetes and metabolic diseases, is not well understood. One study reported that senescent pericytes contribute to impaired barrier function in an *in vitro* model of BBB (Yamazaki et al., 2016). There are no direct studies on how diabetes impacts senescence in pericytes. However, Liu et al. have shown that increased ROS and AGEs production concomitant with pericyte dysfunction in cerebral blood vessels from old diabetic rats and cultured pericytes grown in high glucose conditions (Liu et al., 2021). We have shown reduced pericyte coverage of microvascular endothelial cells in the brains of diabetic rats, which also displayed cognitive deficits (Prakash et al., 2012; Prakash et al., 2013).

Aging research has shown how vascular smooth muscle cell senescence plays a very important role in the context of cardiovascular dysfunction due to their contribution to vascular calcification. Vascular calcification, in which there is a pathological deposition of minerals, leads to vessel stiffening. At a cellular level, this is an outcome of the change in VSMC phenotype from a healthy state to a unique senescent as well as pro-calcification phenotype (Burton et al., 2010), and hyperglycemia has been shown to play a role in this transition (Chen et al., 2006). The increase in ROS and mitochondrial dysfunction, which are hallmarks of the SASP, has been shown in the cerebral VSMCs grown in high glucose conditions (Guo et al., 2020). Also, a direct link between VSMC premature senescence and diabetic condition has been shown in which rutin, a flavonoid drug, was found to decrease the atherosclerotic burden along with the VSMC senescence burden in a mouse model of diabetic atherosclerosis. It was further confirmed by the same group that this was due to decreased oxidative stress-induced senescence in VSMCs *in vitro* (Li Y. et al., 2018). An important study has shown that a decrease in SIRT1 levels in VSMCs due to hyperglycemic conditions *in vitro* is linked to a parallel increase in senescent markers as well as the switch to a pro-calcification phenotype indicated by increased RUNX2 levels. Pharmacological activation of SIRT1 has reversed the harmful senescent/pro-calcification phenotype in the VSMCs indicated by a decrease in RUNX2 expression (Bartoli-Leonard et al., 2019). Collectively, these past reports provide evidence for increased senescence in mural cells in diabetes.

### 3.4 Glial Senescence in Diabetes and Metabolic Diseases

About 50% of the cells in the brain are glial cells: astrocytes, microglia, and oligodendrocytes. All the three cell types play very important roles in brain function. Astrocytes support neurons by providing nutrients like lactate and growth factors, contributing to the BBB stability and integrity, communicating signals in neurovascular coupling, and doing more. Microglia, on the other hand, are the resident immune cells of the brain. Oligodendrocytes, which arise from oligodendrocyte progenitor cells (OPCs), are the myelin-producing cells of the CNS. Two seminal studies have reported that glial cells undergo senescence, and this is associated with the development of cognitive impairment in two different models of dementia (Bussian et al., 2018; Zhang et al., 2019). Furthermore, their removal by genetic and pharmacological approaches prevents cognitive deficits. Bussian and colleagues reported the accumulation of senescent astrocytes and microglia in the brains of *MAPT* ^*P301S*^ *PS19* mice (Bussian et al., 2018). When these cells were cleared using the *INK-ATTAC* transgene approach or with a first-generation senolytic, neurovascular pathologies and cognitive deficits were alleviated. Zhang and colleagues reported senescence in OPCs but not in other glial cells including mature oligodendrocytes, in the brains of patients with AD as well as in the APP/PS1 transgenic mouse model (Zhang et al., 2019). They also showed that pharmacological removal of these cells prevents cognitive impairment. While these studies and others clearly show senescence in glial cells and suggest removal of senescent cells by senotherapeutics is promising, there are no studies specifically conducted in models of diabetes. We refer the readers to excellent articles for a detailed review of glial senescence in neurodegenerative diseases (Cohen and Torres, 2019; Han et al., 2020; Ungerleider et al., 2021).

## 4 Concluding Remarks

The field of senescence has evolved significantly since its discovery about 60 years ago. It is not just a matter of aging. The vast heterogeneity of senescence phenotype and SASP diversity is now being recognized. The International Cell Senescence Association recently published recommendations to define key molecular and cellular features of senescence to drive the field forward (Gorgoulis et al., 2019). Senotherapeutics have emerged as a strategy to promote healthy aging and prevent age-related disease. These include senolytics to remove senescent cells, senomorphics to block SASP factors, and senoinflammation to enhance immune system-mediated clearance of senescent cells (Kim and Kim, 2019). As discussed above, diabetes promotes premature metabolic aging. Our understanding of the role of neurovascular unit senescence in the increased risk of cognitive complications in diabetes is poor. As modern tools are being incorporated into senescence research in the context of the new guidelines, we emphasize the need for further research to investigate the role of neurogliovascular unit senescence in models of diabetes and metabolic diseases.
